# Supplementary material for: Resolving the full spectrum of human genome variation using Linked-Reads
Source: Genome Res. 2019 Apr;29(4):635–45. doi: 10.1101/gr.234443.118 (PMC6442396; doi:10.1101/gr.234443.118)
Supplement: Supplemental Material [file supp_gr.234443.118_Supplemental_longranger-2.2.zip › longranger-2.2.tar.bz2!/lr2.2-public/lib/stan/cmdstan/stan/doc/api/html/math_2matrix_2log__sum__exp_8hpp_source.html]

Stan: src/stan/math/matrix/log\_sum\_exp.hpp Source File


|  |  |
| --- | --- |
|  | Stan  2.5.0  probability, sampling & optimization |


- Main Page
- Related Pages
- Namespaces
- Classes
- Files

- File List
- File Members

All Classes Namespaces Files Functions Variables Typedefs Enumerations Enumerator Friends Macros Pages

- src
- stan
- math
- matrix

log\_sum\_exp.hpp

Go to the documentation of this file.

1 #ifndef STAN\_\_MATH\_\_MATRIX\_\_LOG\_SUM\_EXP\_HPP

2 #define STAN\_\_MATH\_\_MATRIX\_\_LOG\_SUM\_EXP\_HPP

3

4 #include <limits>

5 #include <vector>

6 #include <boost/math/tools/promotion.hpp>

7 #include <stan/math/functions/log1p.hpp>

8 #include <stan/math/matrix/Eigen.hpp>

9

10

11 namespace stan {

12

13  namespace math {

14

28  template <int R, int C>

29  double log\_sum\_exp(const Eigen::Matrix<double,R,C>& x) {

30  using std::numeric\_limits;

31  using std::log;

32  using std::exp;

33  double max = -numeric\_limits<double>::infinity();

34  for (int i = 0; i < x.size(); i++)

35  if (x(i) > max)

36  max = x(i);

37

38  double sum = 0.0;

39  for (int i = 0; i < x.size(); i++)

40  if (x(i) != -numeric\_limits<double>::infinity())

41  sum += exp(x(i) - max);

42

43  return max + log(sum);

44  }

45

46  }

47 }

48

49 #endif

stan::math::log\_sum\_exp

boost::math::tools::promote\_args< T1, T2 >::type log\_sum\_exp(const T2 &a, const T1 &b)

Calculates the log sum of exponetials without overflow.

**Definition:** log\_sum\_exp.hpp:49

stan::math::exp

Eigen::Matrix< T, Rows, Cols > exp(const Eigen::Matrix< T, Rows, Cols > &m)

Return the element-wise exponentiation of the matrix or vector.

**Definition:** exp.hpp:18

stan::math::max

double max(const double a, const double b)

**Definition:** max.hpp:7

Eigen.hpp

stan::math::log

Eigen::Matrix< T, Rows, Cols > log(const Eigen::Matrix< T, Rows, Cols > &m)

Return the element-wise logarithm of the matrix or vector.

**Definition:** log.hpp:16

stan::math::sum

double sum(std::vector< double > &x)

**Definition:** sum.hpp:10

log1p.hpp

stan::agrad::log

fvar< T > log(const fvar< T > &x)

**Definition:** log.hpp:15

stan::agrad::exp

fvar< T > exp(const fvar< T > &x)

**Definition:** exp.hpp:16


---

[ Stan Home Page ]

*© 2011–2014,
Stan Development Team.*
